# Current Assessment and Future Perspectives on Phytoremediation of Heavy Metals

**DOI:** 10.3390/plants14182847

**Published:** 2025-09-12

**Authors:** Aniruddha Acharya, Nacer Bellaloui, Andrej Pilipovic, Enrique Perez, Miller Maddox-Mandolini, Hania De La Fuente

**Affiliations:** 1 Department of Biological & Earth Sciences, Arkansas Tech University, Russellville, AR 72801, USA; 2 Crop Genetics Research Unit, Agriculture Research Service, United States Department of Agriculture, 141 Experiment Station Road, Stoneville, MS 38776, USA; nacer.bellaloui@usda.gov; 3 Natural Resources Research Institute (NRRI), University of Minnesota, Duluth, MN 55811, USA; pilip015@d.umn.edu; 4 Department of Biology & Chemistry, Texas A&M International University, Laredo, TX 78041, USA; enriqueperez@dusty.tamiu.edu (E.P.); haniadelafuente@dusty.tamiu.edu (H.D.L.F.); 5 College of Nursing, Health & Sciences, Delta State University, Cleveland, MS 38733, USA; mmandolini@deltastate.edu

**Keywords:** heavy metals, phytoremediation, environment, agriculture, technology

## Abstract

Heavy metals such as zinc, manganese, nickel, cobalt, copper, iron, and molybdenum are required in minute quantities to maintain optimal biological functions. However, most other heavy metals are not required for living cells; thus, their accumulation within cells and tissues poses a serious threat to human health and the environment. Phytoremediation can offer a safe, inexpensive, and ecologically sustainable technique to clean habitats contaminated with heavy metals. Several herbaceous and woody plants have been identified and utilized as potential candidates for phytoremediation, and the technique has transformed from being in the formative stage, where it was confined to laboratories and greenhouses, to becoming a widely applied technology involving field trials across the globe. However, recently, several field studies have shown promising results that can propel the large-scale implementation of this technology at industrial sites and in urban agriculture. The commercialization of this technique is possible if an interdisciplinary approach is employed to increase its efficiency. Identification of the genetic mechanisms and the cell signaling pathways involved in phytoremediation may support biotechnological intervention through OMICS and CRISPR approaches, resulting in an improvement in the efficiency of the process. This review presents a comprehensive overview of phytoremediation with a focus on the current assessment and future perspectives of the technique. It illustrates the concept of phytoremediation, the ecological and commercial benefits, and the types of phytoremediation. The candidate plants and factors that influence phytoremediation are discussed. The physiological and molecular mechanisms, along with perspectives on the future of the technique, are also illustrated. This review presents clear and updated information on this rapidly evolving technology, thus providing the public and private sectors with essential knowledge on phytoremediation mechanisms. This may assist in policy development for the management of heavy metals while accelerating the development of transgenic plants or other tools that might be more efficient in phytoremediation.

## 1. Introduction

### 1.1. What Are Heavy Metals?

The term “heavy metals” has not been clearly defined in numerous scientific studies, leading to confusion; however, there is consensus that they must have a density above 5 g cm^−3^, along with an atomic number greater than 20 [1]. Though the metalloid arsenic and the non-metal selenium do not strictly adhere to the chemical definition of heavy metals, they are widely included in this category due to toxicological relevance in the literature pertaining to environmental science and pollution and due to their detrimental effects on living organisms [1]. In this review, we clustered the metalloid As (arsenic) and nonmetal Se (selenium) in the heavy metal category for ease of discussion and to imply their biological ramifications rather than chemical exclusivity.

### 1.2. Sources and Risks of Heavy Metals

The accumulation of high amounts of these elements in agricultural soil, air, water, and human habitats is often referred to as heavy metal pollution. The sources of such pollutions can be both from natural and anthropogenic activities (Table 1), such as the weathering of rocks, volcanic eruptions, mining, industrial waste, agricultural runoff, fossil fuels, oil spills, pesticides, and the military industry [2,3,4,5,6,7]. Due to the fundamental nature of elements, they cannot be further degraded by biological processes; thus, they remain unaltered in the environment for long periods of time while their accumulation continues due to anthropogenic activities. The results of these activities pose environmental hazards and global health concerns [8,9,10]. They are sequestered by plants and living organisms, and their concentrations increase in tissues as they move to higher trophic levels; this is referred to as biomagnification [11].

### 1.3. Effect of Heavy Metal on Plants

Heavy metals have a high atomic weight, and most of them are toxic to biological systems [3,12], though some are considered useful and necessary for the completion of the life cycle of plants. Metals such as As, Ag, Cr, Cd, Pb, Hg, and Se have no physiological functions in plants [13], while others, such as Fe, Cu, Zn, Mo, and Mn, are required in small amounts for the optimal growth and development of plants [14,15,16]. One of the adverse effects of heavy metals is the production of free radicals or reactive oxygen species within cells that causes oxidative stress, leading to damage of biomolecules, cell membranes, cellular organelles, and cell death [17,18]. They adversely affect several biochemical, cellular, and physiological processes in plants, which results in chloroplast damage; reductions in chlorophyll content, the photosynthetic rate, transpiration rate, stomatal conductance, and relative water content in leaves and xylem vessels, thus negatively affecting cell growth and enlargement and leading to the inhibition of plant growth [19,20]. Therefore, the accumulation of heavy metals in the ecosystem is a serious health and environmental hazard [21,22].

### 1.4. What Is Phytoremediation?

Phytoremediation involves the use of plants to clean soil and water habitats that are contaminated with heavy metals, herbicides, petroleum products, pharmaceuticals, and other compounds that are considered harmful to the environment. It should be noted that phytoremediation is often mistakenly conflated with bioremediation; however, they are not identical. Bioremediation involves environmental remediation through the use of biological entities such as plants and microbes. Several characteristics of microalgae, such as their rapid growth rate, cost-effective production, and metabolic diversity, offer significant advantages for the bioremediation of heavy metals. Recently, microalgae such as *Chlamydomonas* have been used for the bioremediation process and for the production of bioproducts [23]. However, phytoremediation exclusively refers to the use of plants for the reclamation of water and soil environments that are contaminated due to anthropogenic activity. This review illustrates the current assessment and future perspectives of this emerging technology and informs the reader about several aspects of phytoremediation, such as plant types, laboratory and field studies, categories of phytoremediation, the molecular basis, biotechnological approaches, and future prospects.

### 1.5. Methodology of the Review

Google Scholar was used to search for the scientific literature related to phytoremediation. The initial timeline set for the search was from the year 1989 to the year 2000. Seminal papers during this timeline were selected, and during this time period, phytoremediation was still a new concept. Following this, the next search was from the year 2001 to the year 2022, and a significant amount of scientific literature was selected and utilized from this timeline. For both searches, the query words used were “phytoremediation” and “heavy metals”. The first draft of this manuscript was prepared in late 2022, and thus, from the year 2022 to the current year (2025), the manuscript was modified on the recommendation of several reviewers, and unique perspectives—such as phytoremediation using woody plants, the role of microbes, field studies, gene editing, circular economy, and future prospects—were explored. In each of the searches, the word “phytoremediation” was used along with respective terms such as “phytoremediation” and “field studies”. No exclusion criteria apart from the timeline stated above were applied while searching in Google Scholar.

## 2. Phytoremediation: An Overview

### 2.1. Advantages of Phytoremediation

Phytoremediation technology has several benefits; thus, it has gained momentum over the past two decades. It is a noninvasive alternative to complementary technologies such as soil excavation, soil washing, and soil burning for the decontamination of polluted sites. It is an economically feasible technology, as it is performed by autotrophs using solar energy and does not require extra space or the installation or maintenance of any specialized instruments [24]. Physical and chemical-based soil remediation methods, which mainly involve soil removal and burial at a prohibitive high price, are not financially feasible [25]. It is estimated that phytoremediation is significantly less expensive than engineering-based remediation methods, including soil washing, and is a financially manageable technique for long-term applicability. An estimate indicates that investment in phytoremediation could be recovered within 7 years [26]. The process can be utilized to harvest metal-rich plant tissues, which have high commercial value. The process of phytoremediation is considered efficient and versatile because several elements, inorganic and organic pollutants, can be removed from solid, liquid, and gaseous substrates. The process can be utilized for the in situ recovery of vast areas of land, water, and air that have been contaminated with pollutants and can be applied for a sustained period. Despite large investments, the physical and chemical methods of remediation can induce secondary pollutants in the recovery site and can cause significant changes in soil properties and soil microflora. Contrary to that, the process of phytoremediation is eco-friendly and is considered an efficient land management strategy as it reduces soil erosion and improves soil quality and soil organic content [27]. Finally, the process of phytoremediation can be of aesthetic value if ornamental plants or flowering plants are used in the process [28]. The recent advances in molecular biology and gene editing, along with the identification of several ion transporters, can propel the advancement and commercialization of phytoremediation as a sustainable technology for heavy metal remediation [29,30].

### 2.2. Phytoremediation Systems

**Figure 1:**
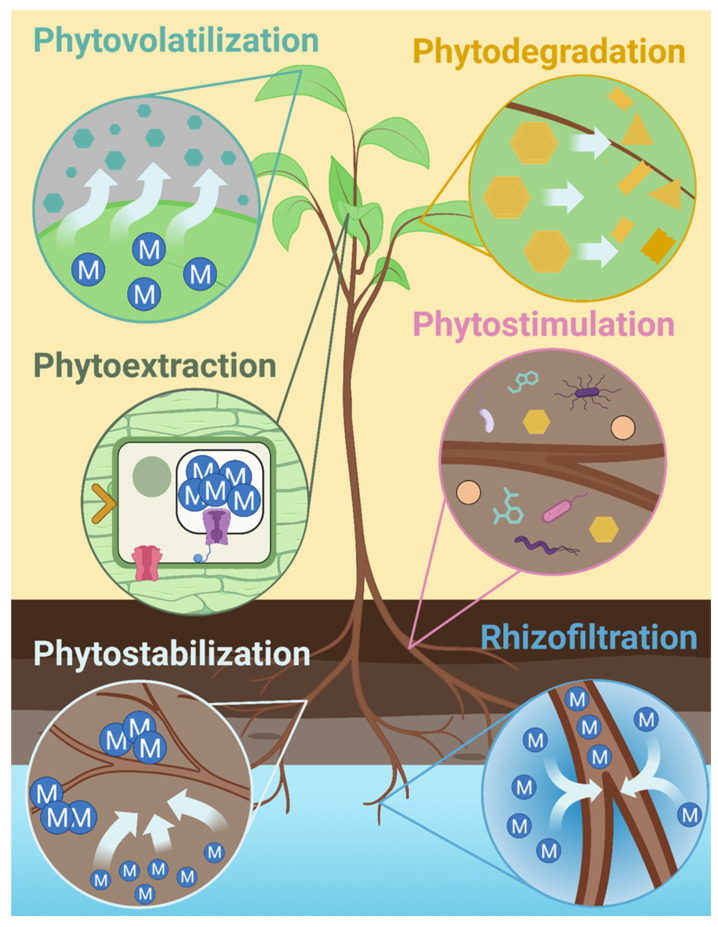
The different mechanisms of phytoremediation of heavy metals include phytoextraction, rhizofiltration, phytodegradation, phytovolatilization, phytostimulation, and phytostabilization. Though these processes are mechanistically different, they share a common target of recovering soil and water from pollutants. Images were created with BioRender.com by Enrique Perez on 6 July 2023 with agreement number UC25KSU74B.

Phytoremediation can be categorized into several types (Figure 1) based on the target material and its fate [31,32]. In the current review, we mechanistically segregated these categories, though many of them have significant overlap [33]. Phytoaccumulation, also referred to as phytosequestration or phytoabsorption, is a process where elements or compounds are sequestered from the soil or water and translocated to the above-ground parts of plants [34]. Phytoextraction or phytominning is the process of extracting metals of high commercial value from harvestable parts of hyperaccumulators. This is a commercially promising technology for mining of high-value metals from polluted environments. The efficiency of the process depends on the chemical properties of the soil, the chemical state of the metal, and the plant species involved [35]. Phytofiltration, also known as rhizofiltration, is the process of cleaning polluted water using plants or their roots. Several elements, radioactive chemicals, and other pollutants can be cleaned from waterbodies by utilizing this technique. Several aquatic plants or terrestrial plants with robust root systems can be utilized for this process. However, high operational cost and the limitations pertaining to the applicability of the process to only a small volume of water have been a major bottleneck of this technique [36]. Halophytic plants are better adapted to saline environments compared to their glycophytic counterparts and can be used to decontaminate saline soil and water environments via the process of phytodesalination [37]. Phytostabilization or phytoimmobilization is the process of employing certain plants to stabilize or reduce the bioavailability of pollutants in the soil; however, the process does not necessarily eliminate pollutants from the soil. Certain elements can be made bioavailable by plant exudates and microbial activity that lowers soil pH in the rhizosphere. However, the leaching of such elements into groundwater or their accumulation in the food chain can be reduced through phytostabilization, where the elements can be adsorbed, precipitated, complexed, and stabilized in the rhizosphere. Thus, phytostabilization transforms toxic compounds into a less toxic form and prevents pollution by reducing their bioavailability [38]. Biotransformation is a process by which certain elements, such as chromium, arsenic, and selenium, are incorporated into organic compounds, thus transforming them from their toxic chemical forms to less toxic organic compounds. Certain plants employ biotransformation either through organic conversion or through chemical reduction of these elements. An example of biotransformation is found in the species *Astragalus*, where excess selenium is converted to nonprotein amino acids such as methylselenocysteine and selenocystathionine [39,40]. Phytodegradation is the enzymatic degradation of organic pollutants by plants and is increasingly being used for reclaiming polluted sites [41,42]. Phytovolatilization involves the use of plants to reduce the bioavailability of pollutants in soil by transforming them into volatile forms that transpire into the atmosphere. Organic and inorganic compounds can be converted into volatile forms that can diffuse through plant tissues and transpire through leaves. The volatilization of selenium has been thoroughly studied, where selenium is converted to the amino acids selenocysteine or selenomethionine, which can be methylated to a volatile compound called dimethylselenide and thus transpire into the atmosphere [43,44]. Phytovolatilization has been applied both naturally and through genetic engineering to remove mercury from soil and convert it into a less toxic and volatile form. Genes such as *merA* and *merB* are widely studied for such processes; however, there are concerns raised about the safety of the technology [45,46,47]. Phytostimulation is a process by which microbes are used to enhance plant physiology and improve stress tolerance in plants. Several bacteria, cyanobacteria, and fungi have been used as phytostimulants to improve plant function and the uptake of heavy metals in plants [48,49].

## 3. Heavy Metal Phytoremediation

### 3.1. Mechanism of Heavy Metal Phytoremediation

Certain enzymes in plants, such as nitrate reductase, contain heavy metals such as molybdenum (Mo). In plants, some heavy metals are required in trace amounts; however, most of them can disrupt cellular homeostasis. Thus, plants employ various strategies to counter it. Plants and microbes can interact in the rhizosphere to reduce the bioavailability of toxic ions, or plants can compartmentalize them in organelles, cells, and tissues that are not photosynthetic in nature. On the contrary, hyperaccumulating plants have evolved to withstand high concentrations of such metals in their tissues and employ chemical, biochemical, microbial, cellular, and molecular mechanisms to achieve such objectives [50,51]. Chemical chelating agents such as EDTA and citric acid, when applied to soil, enhance the phytoremediation of heavy metals. The chelating agents form a metal–chelate complex that can be absorbed, transported, and accumulated within plants. Besides the chemical chelating agents that can be applied externally to the soil, several plants can produce endogenous compounds with metal-chelating properties [52,53]. Phytosiderophores and peptides such as phytochelatins and metallothioneins are biomolecules that can chelate metals and are produced in plants in response to metal deficiency. The relation between metal chelating peptides and metals is highly specific, and such interactions help to reduce phytotoxicity and maintain cellular homeostasis [54,55]. Several metabolites; metal–proteins; chelating compounds; and enzymes such as metallothioneins, phytochelatins, glutathione-S-transferases, and phytosiderophores have been utilized to enhance the process of phytoremediation [56,57,58]. Metallothioneins are low-molecular-weight, cysteine-rich, cytosolic proteins that can bind to several metals, such as cadmium, copper, zinc, and arsenic. They reduce the harmful effects of such metals in plant cells through chelation, sequestration, detoxification, and metal homeostasis [59,60]. Genes encoding metallothioneins have been identified in several plants, including *Arabidopsis*, sugarcane, and rice, and have been overexpressed in plants such as tobacco and *Arabidopsis*, resulting in heavy metal tolerance [61,62,63,64]. Metallothioneins are divided into several sub-groups based on the position of cysteine residue in their polypeptide and are increasingly becoming an important area of research in phytoremediation [65]. Phytochelatins are enzymatically synthesized low-molecular-weight polypeptides that are mostly involved in metal detoxification, metal homeostasis, and abiotic stress and are found in plants, animals, and fungi [66,67]. They are rich in cysteine and are structurally related to glutathione synthetase. There are several structural variations of phytochelatins; however, they chelate metals through their thiol groups and are synthesized by the enzyme phytochelatin synthase [68,69]. The overexpression of enzymes related to phytochelatin biosynthesis in *Brassica* was reported to result in higher levels of phytochelatins in transgenic plant tissues, resulting in higher cadmium tolerance of the plant. Glutathione-S-transferases are involved in phytochelatin biosynthesis and have been linked with copper, aluminum, and arsenic tolerance in plants [70,71,72]. Besides heavy metal tolerance and detoxification, they have been reported to confer defense against oxidative stress in plants and yeast due to their thiol group and have been reported to play a role in modulating gene expression [73]. Phytosiderophores are low-molecular-weight metabolites released by roots in response to iron deficiency and can chelate iron molecules. However, they have also been reported to chelate toxic elements and thus can be an ideal candidate to augment phytoremediation [74].

Ions in a soil solution enter the plant root cells through membrane-bound transporters. The soil solution encounters barriers such as exodermis and endodermis before reaching the vascular tissues of plant roots. The metal can be stored in roots or can be transported to the above-ground parts of the plant through the vascular tissues. To maintain homeostasis, most heavy metals are transported to the vacuoles and thus are compartmentalized within a cell. The soil surrounding the roots is a site of increased microbial activity. Such activity results in enzymatic reactions that can detoxify pollutants and reduce the bioavailability of toxic ions. Plant growth-promoting bacteria (PGPR) can also influence the reduction of heavy metal stress in plants by enhancing nitrogen fixation and phosphorus absorption by roots [75]. Identification of the molecular machinery involved in the acquisition, transport, storage, and metabolism of heavy metals might lead to an understanding of the detailed mechanism of phytoremediation. Such investigations might accelerate the development of transgenic plants that might be more efficient in phytoremediation. Several proteins, such as ZIP (ZRT-IRT-like proteins), NRAMP (naturally resistant associated macrophage protein), HMA (heavy metal P1B-type ATPases), and MTP (metal tolerance protein), which belong to the category of CDF (cation diffusion facilitator) protein, have been associated with heavy metal tolerance in plants. Increased expression of ZIP proteins was associated with higher acquisition, transport, and accumulation of Zn in plants. The NRAMP proteins localized in the plasma membrane and tonoplast are associated with Fe transport in plants. The HMA proteins localized in the plasma membrane, vacuole, and chloroplast are associated with heavy metal homeostasis [76,77]. Members of the ZIP transporter family, such as ZRT-IRT-like protein, are involved in Zn and Cd transport. Gene expression studies involving several genes of the ZIP and IRT-like proteins confirmed their role in Zn and Cd transport in plants [78,79]. In rice, the tissue-specific localization of ZIP transporters such as *OsZIP5* and *OsZIP9* and their role in Zn and Cd uptake have been confirmed through mutation experiments [80,81]. Genome-wide identification and transcriptome analysis of genes of the HMA family indicated their role in Cd stress in plants [82]. Cadmium resistance in plants has been attributed to the high transcription levels of genes of the UPS (ubiquitin proteosome system) pathway that might assist in the degradation of misfolded proteins [83]. Arsenic resistance in plants has been related to transmembrane transporters such as ACR3 (arsenical compound resistance 3), while arsenic transport is related to arsenite effluxers such as PvAsE1 [84,85]. The role of several high-affinity Pi transporters in arsenic transport in plants has been confirmed [86]. Nickel hyperaccumulation and its sequestration in vacuoles of plant cells is attributed to IREG/Ferroportin transporters [87] and NcIREG2 transporter [88], while transporters such as OsNramp5 and OsMTP9 are related to Mn transport in plants. The other transporters and proteins involved in Mn transport are Nramp5 and MTP8.1 [89,90]. The role of the gene *OsMYB45* has been identified, and it confers cadmium resistance in rice [91]. Several enzymes in plants require molybdenum, and non-specific molybdate transporters in several plants have been identified, with MOT1 being a putative transporter for molybdenum in higher plants [92].

### 3.2. What Are Hyperaccumulators?

Certain plants can sequester ions from the soil and store them in their cells at concentrations that are much higher than their counterparts can accumulate in their natural habitat. Cells require several ions in certain threshold concentrations to support optimal cellular metabolism. Thus, the accumulation of ions within cellular organelles and cytoplasm is an essential physiological process and is required to maintain metabolic activities and homeostasis [93,94]. Ions or elements are utilized in several metabolic pathways to maintain the structural identity and functionality of cells [95]. Certain plants in nature have the capacity to accumulate specific elements in their tissues in unusually high concentrations that are not found in tissues of most other plants in similar environments. These plants are called hyperaccumulators, and the relation between hyperaccumulators and the element they accumulate is highly specific [96]. There are several plants that have been identified in nature as hyperaccumulators, and they can sequester metals through roots and translocate them to aboveground parts, such as stems and leaves, where they can accumulate at concentrations that are a few magnitudes higher than non-accumulators [97]. Such high concentrations are considered toxic or even lethal for most plant species, but cannot cause any physiological damage to hyperaccumulating species (Table 2).

Hyperaccumulators are often utilized to sequester specific elements to reclaim habitats, while in other cases, they are utilized to sequester commercially important elements such as gold, platinum, and palladium by a process called phytomining. Thus, the process of phytomining and phytoremediation overlaps mechanistically but has different objectives (commercial vs. environmental). As mentioned before, bioremediation is used in a larger context where the use of any biological organisms, including plants, is considered for the removal of hazardous compounds from the environment [118,119]. Phytoaccumulation can be defined as a phytoremediation technique where hyperaccumulating plants sequester metals through roots and translocate them to different plant tissues for accumulation. On the contrary, phytostabilization is another mechanism of phytoremediation that utilizes plants to prevent the spread of pollutants and mitigate their harmful effects. The process involves in situ treatments of contaminated soils, sediments, landfills, agricultural fields, mines, military, and industrial sites [120]. Heavy metals from different wastes, such as pesticides, herbicides, fertilizers, and effluents, cannot be degraded; however, they can be immobilized and can be harvested through plant tissues [121]. The primary goal of phytostabilization is to reduce the mobility of contaminants by sequestering them in roots or immobilizing them in the rhizosphere. The process can be enhanced through the addition of soil amendments and continuous soil monitoring [122].

### 3.3. Factors That Influence Phytoremediation

The ease of cultivation, maintenance, and harvesting of plant materials from a hyperaccumulating plant are the most essential parameters that need to be considered when selecting candidate plants for phytoremediation. The selected plant species must be easily cultivable, maintained, and the heavy metal-accumulating plant parts must be easily harvestable for economic feasibility. The use of native plant species should also be prioritized, as they are naturally adapted to local conditions and pose lower ecological risks. A plant that is hardy, can grow in marginal soil, and accumulates most of the sequestered pollutants in aerial organs such as stems and leaves can serve as a good candidate plant for phytoremediation. However, if the plant is aquatic, then harvesting the whole plant is relatively easier compared to its terrestrial counterpart. The extraction of elements of high commercial value is one of the primary goals of phytomining. Thus, the process of phytoremediation is economically competitive over physical and chemical methods if the extraction of elements from the harvested plant tissues is easily achievable. Plants can get infected by an array of pathogens; thus, the susceptibility of the candidate plant to pathogens should be considered before cultivating it on a contaminated site. From an ecological perspective, caution should be employed such that the plants used for phytoremediation should not be a source of food for herbivores, as it might lead to biomagnification of pollutants through the food chain. The genetic make-up of the plant to efficiently sequester, translocate, and accumulate toxic ions or detoxify pollutants is an important factor for phytoremediation. Unfortunately, most of the plants that can accumulate high levels of heavy metals have a slow growth rate and low biomass [123,124]. Plants that can grow on marginal soil with limited water supply and are resistant to abiotic stress while accumulating high amounts of toxic metals are ideal for phytoremediation. Genetic engineering can be used to create transgenic lines with such properties. The use of hormones and chelating agents has been reported to augment phytoremediation; thus, a combination of physiological, chemical, and genetic techniques can be used to increase the efficiency of the process [119,125,126]

## 4. Selecting an Ideal Candidate for Heavy Metal Phytoremediation

### 4.1. Plant Ideotype

An ideal candidate for phytoremediation must have several desirable characteristics; however, it is difficult to find a plant that has all those traits [127,128]. Unless aquatic, the plants should have a deep and highly branched root system. Such a robust root system can interact with a large volume of soil and thus can sequester ions from it. The deeper root system will allow the extraction of solutes from deeper regions of soil where the metal might have leached. However, if the candidate plant is aquatic, the deeper and branched root system, though important, will not be as important a factor as in the case of terrestrial plants because waterbodies have a more uniform gradient of ion concentration than the terrestrial environment. Thus, hyperaccumulating aquatic plants can sequester specific ions from contaminated water bodies even if they lack highly branched and deep root systems. The interaction of plant roots with the biotic and abiotic factors in the rhizosphere plays a major role in the bioavailability of the elements. Thus, the nature of these interactions and soil chemistry are factors that need to be considered before selecting a hyperaccumulator for in situ phytoremediation [129,130]. Candidate plants for phytoremediation should have the capacity to tolerate high ionic concentrations in their growing environment. Even at small concentrations, toxic elements, pollutants, and xenobiotic compounds have detrimental effects on plant physiology. Thus, an ideal candidate plant for phytoremediation must have the capacity to withstand high concentrations of these elements in their contaminated habitats [131]. They must be able to sequester and compartmentalize the elements within their cell while maintaining ionic homeostasis.

Besides the robust root system, another critical factor for an ideal candidate for phytoremediation is the growth rate of the plant. A rapid growth rate is desirable so that the contaminated site can be cleaned in a relatively realistic time frame. Plants can take a few months to years or even decades to attain maturity and complete their life cycle. Some plants, such as *Sequoiadendron*, can grow for centuries. Phytoremediation directly depends on the physiology, growth, and development of the candidate plants [132]. Biological processes such as growth and development are much slower than compared to chemical and mechanical processes [133]. As phytoremediation is a biological process, the decontamination of pollutants is much slower compared to chemical and mechanical processes that can be used for this purpose. So, it is extremely important that the candidate plant selected for phytoremediation has a high growth rate to achieve decontamination of polluted sites within a realistic timeframe. The biomass of the plant is another critical factor that needs to be considered when selecting candidates for phytoremediation. The total amount of elements that can be extracted from contaminated sites via the process of phytoremediation can be calculated by multiplying the concentration of the metal in harvestable parts of the hyperaccumulating plant by its dry mass. A plant having high biomass will thus serve as a more useful candidate than one that has low biomass. Plant biomass is an abundant and renewable resource; thus, a hyperaccumulating plant with high biomass can be repeatedly sown and harvested to decontaminate land and waterbodies from pollutants and toxic metals [134].

### 4.2. Herbaceous Plants Used in Heavy Metal Phytoremediation

Plants that can thrive on high metal concentration are called metallophytes. Some metallophytes are capable of accumulating high concentrations of metals and are classified as hyperaccumulators [135,136]. A large percentage of hyperaccumulators are represented by the *Asteraceae*, *Brassicaceae*, *Cyperaceae*, *Euphorbiaceae*, *Fabaceae*, and *Poaceae* families of plants. There are nearly 500 species from 50 families that are categorized as hyperaccumulators. *Brassica juncea* and *Typha latifolia* L. have been utilized for phytoremediation of selenium [137], while *Pteris vittate* is utilized to decontaminate sites with arsenic contamination [138]. *Helianthus annuus*, or sunflower, is another good candidate for phytoremediation [139]. Grasses such as *Trifolium alexandrinum* have been used for phytoremediation of heavy metals [140]. Among aquatic plants, *Pistia stratiotes*, or water lettuce, has been used to decontaminate manganese pollution in waterbodies [141]. Several aquatic plant species, such as *Azolla*, water hyacinth, and duckweed, are used for phytoremediation of waterbodies due to their high growth rate, high biomass, and adaptation capabilities and can be easily harvested for target elements [142]. Several plants (Table 2), including *Thlaspi*, *Brassica*, *Jatropha*, *Helianthus*, and *Alyssum*, have been investigated for their hyperaccumulation and phytoremediation properties. Genetically modified *Arabidopsis thaliana* and *Nicotiana tabacum* have been used for the phytoremediation of mercury [143,144,145].

### 4.3. Woody Plants Used in Heavy Metal Phytoremediation

Hydroponic experiments in a controlled environment indicated that woody plants such as *Salix matsudana* and *Salix nigra* had high tolerance to cadmium stress and thus can serve as phytoremediation candidates for the reclamation of cadmium-contaminated sites [146]. Hydroponically grown *Salix viminalis* were found to be a good candidate for phytoextraction of cadmium in an iron- and magnesium-deficient environment [147]. Other woody plants, such as black locust, when grown hydroponically, were found to be promising candidates for the phytoextraction of heavy metals such as cadmium, nickel, and lead [148]. In vitro experiments using white poplar, such as *Populus alba* L., yielded promising results with regard to nickel extraction from environments that have low nickel contamination [149]. Greenhouse experiments using poplar and willow plants revealed that they were good candidates for phytoremediation of nitrates from groundwater and sediment [150]. Woody plants, such as poplar and willow trees, were evaluated for their phytoremediation potential in removing heavy metals, including chromium, copper, lead, zinc, nickel, and cadmium. River sediment- and heavy metal-contaminated soil were used in controlled settings of a greenhouse for estimating the phytoremediation capacity of poplar and willow trees [151]. Poplar clones showed promising results in phytoremediation of heavy metals such as nickel, copper, and cadmium from soil [152]. Poplar varieties such as *Populus deltoides* and *Populus × euramericana* were found to be ideal candidates for phytoremediation of cadmium from contaminated soil [153]. Poplar trees were found to be an efficient candidate for phytoremediation when grown in soil that was artificially contaminated with herbicides; diesel; or heavy metals such as cadmium, copper, and nickel [151]. Chelating agents such as citric acid were found to positively impact the growth parameters of *Salix viminalis* in cadmium-contaminated soil [52]. Addition of exogenous citric acid was reported to improve sequestration of cadmium by *Salix viminalis*, *Salix alba*, and *Salix matsudana* from cadmium-contaminated soil while ameliorating the negative effect of the heavy metal on the physiology of Salix plants [20].

### 4.4. Microalgae and Circular Economy

Microalgae are an excellent option for cost-effective removal of heavy metals and organic pollutants from contaminated wastewater. Several characteristics, such as metabolic diversity, rapid growth rate, high biomass, tolerance to several pollutants, and their efficient removal, have encouraged the use of microalgae in phytoremediation. Microalgae such as *Arthrospira platensis* have been evaluated for their role in heavy metal bioremediation of contaminated water. In a controlled lab-based investigation, several heavy metals, such as Cu, Cd, Ni, and Pb, were efficiently recovered by *Arthrospira platensis* cultures from mono- and multi-metal solutions. The high-value metals from the contaminated biomass can be extracted and utilized for valuable commercial products such as catalysts, batteries, paints, and pesticides [154]. The potential of microalgae such as *Chlamydomonas*, *Scenedesmus*, and *Chlorella* in removing radionuclides such as Americium (Am), Cesium (Cs), Cobalt (Co), and Plutonium (Pu) has been investigated [155]. Cyanobacteria have been efficiently used for the removal, recovery, and reuse of heavy metals for the production of silver, gold, copper, zinc, cadmium, and titanium nanoparticles with several commercial applications, thus contributing to the circular economy [156]. Mass cultivation of microalgae in wastewater offers a lucrative option for heavy metal bioremediation besides the production of biofuel, thus propelling circular economic principles of reuse, reduce, and recycle [157]. Microalgae such as *Chlorella*, *Chlamydomonas*, *Microcystis*, *Spirulina*, and *Spirogyra* are used for the removal of heavy metals from the environment due to their abundance and efficiency, besides being an economical option for bioremediation [158]. Following phytoremediation, microalgal and cyanobacterial biomass can be used as biofertilizer for sustainable agriculture, thus promoting the circular economy [159]. The use of phytohormones to promote microalgal growth in heavy metal-contaminated wastewater has been explored [160]. Microalgae cultivated on wastewater from fish processing industries have been utilized for the production of high-value polysaccharides using circular economy approaches [161].

### 4.5. Use of Biotechnology in Phytoremediation

**Figure 2:**
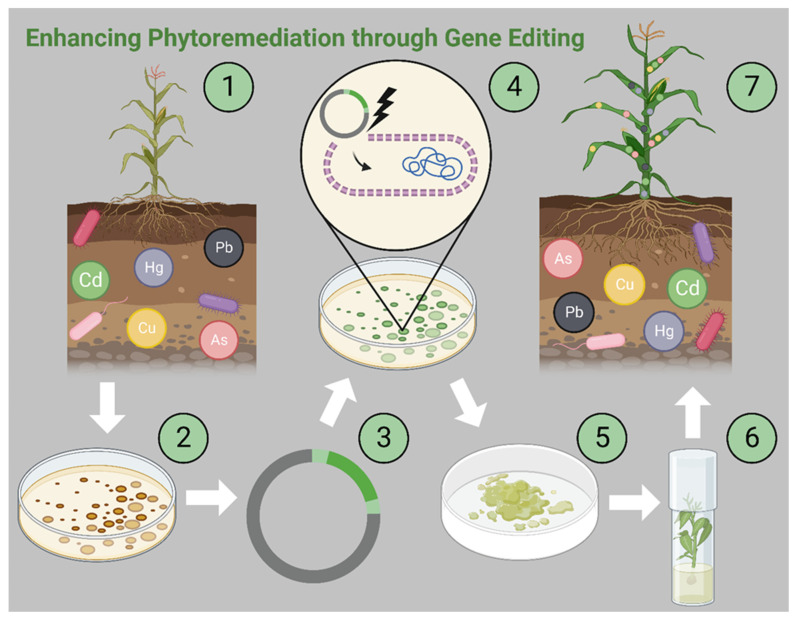
Prospects of enhancing the phytoremediation technology through genetic engineering and genome editing, where microbes and/or plant genes responsible for phytoremediation can be identified and cloned into a vector (1, 2, and 3) for genetic engineering. CRISPR gene editing technology can assist in precise gene manipulation to enhance the phytoremediation process. The gene of interest can be transferred to pluripotent callus cells through gene gun or *Agrobacterium*-mediated transformation (4 and 5). Finally, the genetically modified plant can be regenerated in vitro and transferred to the field (6 and 7). Image was created with BioRender.com by Enrique Perez on 6 July 2023 with agreement number JC25KSTXKE.

The CRISPR (Clustered Regularly Interspaced Short Palindromic Repeats) technology for genome editing is one of the latest advancements in recombinant DNA technology. It supersedes the power and precision of previously known genetic engineering technologies while reducing the time and cost for the generation of mutants and transgenic lines. The significant reduction in DNA sequencing costs and the availability of genome sequences for several plant species have accelerated the use of CRISPR for the modification of plant genomes (Figure 2), using genes from distant species or modulating the expression of endogenous genes [162]. Several mechanisms involved in heavy metal phytoremediation, such as stabilization, sequestration, translocation, accumulation, and transformation, can be enhanced using this technology [163]. Genes encoding metal-binding proteins, such as metallothioneins; metal transporters such as *ZIP*, *MATE*, and *HMA*; and metal chelators such as phytochelatins have been the primary targets to enhance phytoremediation using CRISPR technology. However, manipulation of hormones, root exudates, and pathways involved in oxidative stress has been targeted for detoxification, stress regulation, and phytoremediation [164,165]. Activation, repression, and overexpression of genes are the common mechanisms employed for modulating gene expression [166]. However, it is essential to manipulate several genes of a pathway to obtain desired results in phytoremediation because most of the phytoremediation mechanisms involve a cassette of genes that work in tandem. Thus, the focus should be on manipulating key genes in a pathway rather than focusing on a single gene. CRISPR allows such manipulation; thus, it is increasingly becoming the primary technology for genetic engineering in plants. CRISPR has been utilized to enhance phytoremediation in rice, corn, and poplar with promising results [167]. The technology can be used to create transgenic lines with higher tolerance for toxic ions and enhance the phytoremediation capacity of hyperaccumulators. Gene pyramiding involves the stacking of genes in plants to impart multiple traits of commercial importance and can be utilized to increase biomass and accelerate growth of such hyperaccumulators [168]. Finally, care must be taken to eliminate ecological risks of gene transfer to native flora and biomagnification of heavy metals in native fauna. Field trials of CRISPR-mediated transgenic lines are necessary to gather conclusive data on the safety and wide-scale applicability of this technology.

## 5. Recent Field Studies

*Field Studies on Heavy Metal Phytoremediation*

In the past few years, several field experiments have yielded promising results on heavy metal recovery from contaminated sites through the application of phytoremediation technology. In Southern China, the phytoremediation potential of the chicory plant was investigated for cadmium recovery from contaminated fields [169]. The effect of crop rotation involving rice and chicory plants was also estimated over a period of seven seasons, resulting in a comprehensive four-year study. The exhaustive dataset resulting from such studies indicated that an amount of 407 g of cadmium per hectare was extracted from contaminated paddy fields after a period of four years [170]. In another field study, chicory plants were grown in contaminated agricultural soil to estimate their efficacy for cadmium phytoremediation. A high amount of cadmium was accumulated in chicory leaves, and an average of 320 g of cadmium per hectare was extracted from the contaminated site, thus indicating the phytoremediation potential of chicory plants for cadmium recovery from contaminated agricultural fields [171]. Field experiments involving the estimation of cadmium recovery by *Sesbania cannabina* when intercropped with rice indicate that the plant can be used to reduce cadmium levels in contaminated paddy fields. This results in a reduction in cadmium levels in rice grains to 0.18 mg Kg^−1^; such an amount of cadmium is considered safe for consumption by food safety standards in China [172]. Water-soluble chitosan was used to enhance the phytoextraction of cadmium by *Hylotelephium spectabile* from contaminated soil. It was concluded to be an effective soil amendment for cadmium extraction in fields [173]. Cadmium- and zinc-polluted farmlands were utilized as experimentation sites to investigate the potential of *Chrysanthemum indicum* L. as a candidate plant for phytoremediation. The three-year study concluded that the plants were able to reduce 78.1% of cadmium and 28.4% of zinc from the contaminated sites [174]. In another field study, several cultivars of *Brassica juncea* L. were identified as good candidates for cadmium and lead removal from low to moderately contaminated soils [175]. Field studies involving *Pennisetum* species identified it as an ideal candidate for cadmium and copper removal from contaminated fields [176]. Plants such as sunflower and sesame are known to have high tolerance for metals; thus, the synergistic effect of crop rotation involving sunflower and sesame was performed in field conditions to estimate their cumulative effect on heavy metal phytoremediation from soil. Several elements, such as cadmium, copper, lead, and zinc, were successfully recovered from soil by the phytoremediation process involving sunflower and sesame [177]. Phytoremediation experiments involving field studies indicated that sorghum was an ideal candidate compared to maize and *Atriplex* for the phytoremediation of cadmium, copper, lead, and zinc from contaminated soil [178]. Giant miscanthus was utilized for the phytoremediation of copper, lead, and zinc from abandoned flotation tailings near Serbia. The results indicated that the miscanthus variety was capable of accumulating high amounts of copper, lead, and zinc in its roots [179]. In another multi-year field study in Croatia involving giant miscanthus, it was found that the plant can effectively accumulate copper, zinc, arsenic, strontium, manganese, titanium, iron, and molybdenum, making it suitable for phytoremediation from contaminated sites [180]. A two-year controlled study followed by a three-year in situ experiment indicated that ferns such as *Pteris vittata* L. can be an ideal candidate for phytoremediation of arsenic from contaminated industrial sites. The aerial parts of the plants recorded a high amount, such as 750 mg Kg^−1^, of arsenic accumulation [181]. Industrial landfills were utilized as experimental sites to estimate the phytoremediation capacity of *Salix* species in combination with arbuscular mycorrhizal fungi. Root mycorrhizal associations have been explored to reduce heavy metal bioavailability and to enhance plant growth [182]. Several elements, such as cadmium, zinc, copper, lead, tin, and barium, were recovered, or their concentrations were found to be reduced from contaminated soil, indicating *Salix* to be an ideal candidate for heavy metal phytoremediation [183]. A study investigated the potential of grasses such as *Vetiveria zizanioides* in the removal of toxic metals from industrial wastewater-fed soils. Results indicated that several metals, such as copper, lead, iron, cadmium, and zinc, could be successfully removed from contaminated soil, thus making it an ideal candidate for the development of green space in urban and industrial areas [184]. Besides terrestrial field studies, constructed wetlands offer a nature-based solution for phytoremediation of heavy metals and are becoming increasingly popular due to encouraging results. Compared to the terrestrial environment, the aqueous environment enables the phytoremediation of a greater volume of pollutants, thus being more cost-effective. The diverse population of macrophytes and microbial consortia can improve efficiency in treating wastewater and pollutants from different sources, such as hospitals, domestic waste, and industrial effluents. Several studies indicate the potential of constructed wetlands for large-scale phytoremediation of pharmaceuticals, organic volatiles, toxins, and pollutants, including heavy metals such as chromium and mercury [185,186,187]. Woody plants such as poplar clones showed promising results when they were evaluated for landfill phytoremediation. Poplar trees have been used for phytoremediation buffer systems in Lake Superior and Lake Michigan watersheds [188,189].

## 6. Challenges and Future Perspective

A fundamental understanding of the biological process of phytoremediation at a biochemical and molecular level is essential for the development of genetically engineered hyperaccumulators. Currently, phytoremediation technology is at its formative stage. Though many plants have been identified as hyperaccumulators, most of the studies are limited to superficial analysis of the uptake of heavy metals by a plant species [190]. A quantitative field study to estimate the commercial potential of the technique, as well as studies involving genetic investigation of hyperaccumulation, are essential for scale-up and broader application. Quantitative, biomolecular, and genetic information will transform phytoremediation into a realistic tool for environmental sustainability and economic benefits. The uptake, translocation, compartmentation, and conversion of metal pollutants to less harmful compounds and their fate have been described to a considerable extent; however, the mechanisms involved have not been dissected. The role of microbes dwelling in the rhizosphere and assisting the hyperaccumulators in heavy metal uptake is reported, but the significant and complex interactions that are involved in the process require further investigation [191]. Synthetic communities (SynComs) have been recently used to estimate their potential in cadmium phytoremediation [192]. The mechanisms that allow the biological system of hyperaccumulators to tolerate such elevated levels of heavy metals might have evolutionary origins by which they were able to protect themselves from pathogens and herbivores [193]. The ecological implications of growing hyperaccumulators on a large scale are also important to understand, as the heavy metals that accumulate in these plants can enter the food chain through the consumption of plant parts, potentially posing a long-term health hazard [11].

Several factors have precluded the wide application of phytoremediation technology on an industrial scale. Though the process of phytoremediation is extensively studied, the genetic and molecular mechanism involved in heavy metal accumulation is poorly understood. This has been a bottleneck for the generation of transgenic plants that would efficiently sequester heavy metals from the environment. The other major concern is the genetic mutation that can result in heavy metal-resistant genes and can be transferred to other plants through pollen, causing biological contamination. The involvement of public and government incentives to develop biorefineries for metal extraction might give impetus to the technology. The process of phytoremediation has tremendous potential for mining of high-value metals and improving the environment of contaminated sites. Industrialization, urbanization, fuel consumption, economic growth, and consumer demands across the globe will further advance the demand for sustainable technology such as phytoremediation. The adoption of phytoremediation as a viable option for environmental reclamation in different countries will largely depend on their economy and commitment towards sustainable practices. The development of remote sensors, artificial intelligence, and predictive modeling will further optimize the efficiency of the process.

Future development of the process of phytoremediation is dependent on a detailed understanding of the role of microbes, membrane transporters, and genetic and metabolic networks; estimation of their efficacy in the field; and investigation of the long-term ecological implications. The rhizosphere is a site of interactions between plant roots and microbes, and a significant relationship exists as a large amount of photosynthates is exudated from the roots to the region that is utilized by the dwelling microbes. Thus, it is extremely important to understand the role of microbes in the phytoremediation process [194]. Recent studies indicate the intricate nature of plants, animals, and microbes in improving soil health [195]. The role of membrane-bound transporters in phytoremediation is one of the aspects that needs rigorous studies. Though several ion transporters have been identified and their role has been established, the transporters specifically involved in phytoremediation are not well studied. Since these metals are not required by the plant to complete its life cycle, and in some cases are required in much smaller amounts, their accumulation might be a byproduct of the sequestration of a similar ion that matches their chemical character. Due to chemical similarity, ions can be transported by the same membrane-bound transporters such as calcium/strontium [196] and potassium/rubidium [197]. Thus, it is important to understand the nature and route of transport. The tissue or cell-specific distribution and activity of these transporters can illustrate the pattern of ion accumulation. Recent studies indicate that the distribution of ions in seedling roots is tissue-specific [198,199,200,201,202]. Anatomical structures such as the Casparian strip and chemical complexes such as lignin and suberin play an important role in ion transport regulation; thus, their role from the perspective of phytoremediation and ion transport might be a pertinent topic of investigation. It is important to understand the role of different ion-transport boundaries in apoplastic and symplastic routes of heavy metal translocation. Plant roots are difficult to study and, until recently, have been largely ignored. However, significant genetic potential exists for their robust development through genetic engineering. Such developments will improve phytoremediation technology. The role of the CBL-CIPK signaling pathway and DELLA proteins is of immense interest for biotechnological interventions and the development of a robust root system that can selectively sequester more ions [203]. The development of genetic tools, gene editing technology, and sequencing of nucleic acids has allowed us to understand the metabolic and regulatory networks of several biological processes in plants, animals, and bacteria. Identification of novel genes and enzymatic pathways that confer heavy metal tolerance to hyperaccumulators is important to create transgenic lines of plants with such characteristics and improve their efficiency. The generation of genetic data related to phytoremediation needs to be managed through a database for long-term applications such as storage, retrieval, and modification when needed [194]. Such a database will help future researchers to understand and build on the technology for practical use. Research in phytoremediation has a corollary benefit in space biology. Several missions on Mars and the moon have excited scientists about the scope of colonizing other planets in space. This has resulted in identifying the factors that are needed for the survival of humans on other planets. Among the many, one important factor is a continuous supply of food, and to achieve this, scientists have investigated plant growth in space. Recent reports about the growth of *Arabidopsis* in moon soil and growing plants in the International Space Station have significantly encouraged the possibility of growing plants in space [204,205]. The soil composition on the moon and Mars is not like Earth and has elements in concentrations that are considered toxic for plants. Thus, the knowledge gained through the understanding of heavy metal tolerance in plants will benefit space biology.

Besides plant biology, several disciplines of science, such as chemistry, soil science, microbiology, biochemistry, genetics, ecology, economics, and chemical engineering, can significantly contribute to the improvement in phytoremediation technology. “Omics” technologies, such as genomics, metagenomics, transcriptomics, proteomics, and metabolomics, along with bioinformatics, can be utilized to improve heavy metal phytoremediation. The manipulation of membrane-bound transporters, identification of translocation and compartmentation of ions in plant tissues, management of oxidative stress, and detoxification of heavy metals by caging and rendering them unavailable to the biological system can improve the phytoremediation potential of plants. However, omics technologies have certain limitations. They often identify large numbers of candidate genes, but their functions are not fully understood, and metal tolerance and detoxification are usually controlled by multiple genes. Some genes can be specific for heavy metal uptake, while others can just be a correlation. Though several plants capable of phytoremediation have been identified, the potential of phytoremediation in the large diversity of the plant kingdom remains largely unexplored. With the availability of sequencing technology and bioinformatics, such identification might be possible by investigating similarities in gene sequences and protein products. The technology of phytoremediation can be integrated with similar environmental and economic objectives, such as carbon sequestration and biofuel production [206]. The goal of heavy metal phytoremediation is their accumulation in harvestable plant parts, while the target of carbon sequestration is trapping the atmospheric carbon in plant tissues. Biofuel projects are geared toward the accumulation of cellulosic organic mass in plants that can be utilized for biofuel production. The above-mentioned three technologies have a common goal of environmental sustainability, plant biomass improvement, and understanding of the mechanisms involved in element compartmentation. Identification of genetic traits for phytoremediation, carbon sequestration, and biofuel production [207,208] is the first step that can lead to metabolic engineering of plants with such properties. The past thirty years have seen phytoremediation gaining momentum; however, most of the work has been exploratory and superficial in nature, besides being limited to the laboratory and greenhouse. Unlike the controlled environment of a greenhouse, the field has several confounding factors, such as soil nutrients, soil amendments, soil chemistry, soil particle size, water retention capacity, precipitation, temperature, wind, seasonal pathogens, and competition from other plants for limited resources. Such factors may interact, and it is important to estimate the effect of these factors on phytoremediation. Thus, more numerous and large-scale field trials of hyperaccumulators are important to establish credibility before this technology can be patented and commercialized. Understanding the mechanism of heavy metal acquisition by plant roots is extremely important for phytoremediation research; however, above-ground plant tissues have been mostly investigated for the process. Innovation in technology [209,210] can accelerate the investigation of the below-ground plant root tissues that have largely been ignored, and this could improve phytoremediation technology [211]. A detailed understanding of the process may lead to its integration with agriculture, which can lead to a sustainable environment and better agricultural management practices [212,213,214]. Though this is an applied field of research, progress in this technology can also propel fundamental research on ion transport, compartmentation, and assimilation. The technology of heavy metal phytoremediation is at its early stage of development; however, it holds immense potential for a sustainable environment, agricultural practices, food security, and research related to space biology.

## Figures and Tables

**Table 1:** Bird’s-eye view of heavy metal phytoremediation.

Different Sources of Heavy Metals	Common Heavy Metal Candidates	Phytoremediation Categories	Factors Influencing Phytoremediation
Mining of ores Fossil fuel Volcanic activity Weathering of rocks Industrial waste Fertilizers Herbicides Pesticides Agriculture Anthropogenic activity	As; Ag; Au Cr; Co; Cd Cu; Fe; Hg Mn; Ni; Pb Se; Zn	Phytoaccumulation Phytomining Phytofiltration Rhizofiltration Phytodesalination Phytostabilization Phytovolatalization Phytodegradation Phytotransformation	Robust root system Bioavailability of elements Heavy metal tolerance capacity Growth rate of plant Biomass accumulation Ease of cultivation Maintenance of plants Ease of harvesting Ease of extraction of heavy metal from plant parts Pathogens that can damage the plant Herbivores that can consume the plant parts

**Table 2:** Commonly used plants for investigation of heavy metal hyperaccumulation and/or phytoremediation capacity.

Elements	Plants	Concentrations of Heavy Metals Recovered from Plant Tissues	Mechanism	Type	References
As	*Pteris vittata*	1639 mg kg^−1^	Rhizofiltration	Hydroponic	[98]
As	*Pteris vittata*	1373 mg kg^−1^	Phytoextraction	Field	[99]
As	*Azolla caroliniana*	397 mg kg^−1^	Rhizofiltration	Field	[100]
Cd	*Phytolacca americana*	637 mg kg^−1^	Rhizofiltration	Hydroponic	[101]
Cd	*Tagetes erecta*	346 mg kg^−1^	Phytoextraction	Pot	[102]
Cd	*Nicotiana tabacum*	23 mg kg^−1^	Phytoextraction	Field	[103]
Co	*Nyssa sylvatica*	438 mg kg^−1^	Phytoextraction	Pot	[104]
Cr	*Leptochloa fusca*	93 mg kg^−1^	Phytostabilization	Pot	[105]
Cr	*Brachiaria mutica*	18 mg kg^−1^	Phytostabilization	Pot	[105]
Cu	*Khaya ivorensis*	329 mg kg^−1^	Phytoextraction	Pot	[106]
Cu	*Commelina communis*	1119 mg kg^−1^	Rhizofiltration	Hydroponic	[107]
Hg	*Helianthus tuberosus*	1 mg kg^−1^	Phytostabilization	Pot	[28]
Hg	*Pistia stratiotes*	83 mg kg^−1^	Rhizofiltration	Hydroponic	[108]
Mn	*Schima superba*	62,412 mg kg^−1^	Phytoextraction	Pot	[109]
Na	*Alternanthera philoxeroides*	145,000 mg kg^−1^	Rhizofiltration	Hydroponic	[110]
Ni	*Alyssum murale*	443 mg kg^−1^	Phytoextraction	Field	[111]
Ni	*Bidens pilosa*	305 mg kg^−1^	Phytostimulation	Pot	[112]
Ni	*Leptoplax emarginata*	7864 mg kg^−1^	Phytoextraction	Field	[113]
Pb	*Sedum alfredii*	915 mg kg^−1^	Rhizofiltration	Hydroponic	[114]
Pb	*Arundinaria argenteostriata*	1117 mg kg^−1^	Phytostimulation	Pot	[115]
Zn	*Thlaspi caerulescens*	20,000 mg kg^−1^	Phytoextraction	Pot	[116]
Zn	*Coronopus didymus*	1848 mg kg^−1^	Phytoextraction	Pot	[117]

## Data Availability

No new data were created or analyzed in this study.
